# Draft Genome Sequence of *Rhodobacter* sp. Strain CACIA 14H1, a Heterotrophic Bacterium Obtained from a Nonaxenic Culture of a *Cyanobium* Species

**DOI:** 10.1128/genomeA.01116-13

**Published:** 2014-01-16

**Authors:** Alex Ranieri Jerônimo Lima, Andrei Santos Siqueira, Bruno Garcia Simões dos Santos, Fábio Daniel Florêncio da Silva, Davi Toshio Inada, Clayton Pereira Lima, Jedson Ferreira Cardoso, João Lídio S. G. Vianez-Júnior, Márcio Roberto Teixeira Nunes, Evonnildo Costa Gonçalves

**Affiliations:** a Laboratório de Tecnologia Biomolecular, Instituto de Ciências Biológicas (ICB), Universidade Federal do Pará (UFPA), Belém, Pará, Brazil; b Centro de Inovações Tecnológicas (CIT), Instituto Evandro Chagas (IEC), Ananindeua, Pará, Brazil

## Abstract

Despite their prominent importance, few efforts have been paid to the genomic analysis of heterotrophic bacteria associated with cyanobacteria. Thus, this work presents the draft genome sequence (~3.9 Mbp) of a heterotrophic bacterium (*Rhodobacter* sp. strain CACIA 14H1) recovered from a nonaxenic culture of a *Cyanobium* species.

## GENOME ANNOUNCEMENT

Despite the assumption that cyanobacteria require the presence of heterotrophic aerobic bacteria for growth (1), efforts for genomic sequencing have been almost exclusively directed toward cyanobacteria. On the other hand, the development of new strategies for assembling metagenomic data derived from nonaxenic cultures (2, 3) can be useful for a better understanding and genetic characterization of many of these heterotrophic bacteria.

The draft genome sequence obtained for the heterotrophic bacterium *Rhodobacter* sp. strain CACIA 14H1 was recovered from total DNA extracted from a nonaxenic culture of cyanobacterium *Cyanobium* sp. strain CACIA 14. The isolate was obtained from a water sample collected in December 2010 from the Tucuruí Hydroelectric dam (3°49′55″S, 49°38′50″W), Pará, Brazil. DNA samples were obtained from *Cyanobium* sp. CACIA 14, cultured, and sequenced in two different periods 6 months apart using the GS FLX 454 sequencer (Roche Life Sciences) with a nonpaired library (4). Two sequencing runs were processed, and the reads generated on each run were assembled separately using the gsAssembler software (Newbler version 2.6 package) with the following parameters: minimum read size of 45 bp, minimum overlap of 40 bp, and minimum overlap identity of 90%. In the first run, a total of 660,228 reads were obtained (~255 Gbp), resulting in 3,654 contigs >1 kbp in length (N_50_, 2,140 bp; 89.9% [~7.2 Gbp] with quality score >40). The second run generated 815,325 reads (~356 Gbp) with 3,256 contigs also >1 kbp (N_50_, 37,998 bp; 97.26% [~20.5 Gbp] with quality score >40). The assembled contigs were identified and separated using a metagenomic assembling pipeline (2) for each potential organism. The second-run assembled contigs were used to determine the genome coverage. This approach allowed for the generation of the draft genome sequence of *Rhodobacter* sp. CACIA 14H1, containing 236 contigs (3.9 Mbp), ranging from 4,886 bp to 64,833 bp, with a mean coverage of 15×, an N_50_ value of 20,479 bp, and a G+C content of 66.64%. The pipeline uses 107 hidden Markov models for essential genes present in a single copy in 95% of all bacteria. The contigs contain 104 of these genes, with TIGR00436 in duplicate, which normally occurs this way.

Structural annotation was performed with the PGAAP tool, available on the NCBI website (5), resulting in 3,571 protein coding genes and 40 tRNA genes. The rRNA clusters were predicted by the RNAmmer tool (6). The 16S rRNA sequence (1,413 bp) for *Rhodobacter* sp. CACIA14H1 showed 99% identity with the 16S rRNA sequence of *Rhodobacter* sp. clone P33 (7), while the *rpoB* gene sequence (3,906 bp) revealed 95% identity with the *rpoB* sequence of *Rhodobacter sphaeroides* ATCC 17025 (8).

The draft genome sequence obtained for *Rhodobacter* sp. CACIA14H1 should be useful for a better understanding of the ecological relationships between the heterotrophic aerobic bacteria and cyanobacterial groups, mainly with regard to their association and influence in bacterial growth and production of substances of biotechnological interest.

### Nucleotide sequence accession numbers.

This whole-genome shotgun project has been deposited at DDBJ/EMBL/GenBank under the accession no. AYNO00000000. The version described in this paper is version AYNO01000000.
